# How to disseminate national recommendations for physical activity: a qualitative analysis of critical change agents in Germany

**DOI:** 10.1186/s12961-021-00729-7

**Published:** 2021-05-06

**Authors:** Laura Wolbring, Anne Kerstin Reimers, Claudia Niessner, Yolanda Demetriou, Steffen Christian Ekkehard Schmidt, Alexander Woll, Hagen Wäsche

**Affiliations:** 1 Institute of Sports and Sports Science, Karlsruhe Institute of Technology (KIT), Engler-Bunte-Ring 15, 76131 Karlsruhe, Germany; 2 Department of Sport Science and Sport, Friedrich-Alexander University Erlangen-Nuremberg, Gebbertstraße 123b, 91058 Erlangen, Germany; 3 Department of Sport and Health Sciences, Technical University of Munich, Georg-Brauchle-Ring 60/62, 80992 Munich, Germany

**Keywords:** Physical activity recommendations, Physical activity guidelines, Dissemination strategy, Physical activity promotion, Change agent, Health promotion

## Abstract

**Background:**

Physical activity recommendations are reached by only a small part of the population. A common problem is that research findings on public health-related topics such as physical activity promotion are oftentimes not translated into practice. The involvement of relevant stakeholders, such as change agents (role models, decision-makers, and/or knowledge mediators), is a common strategy to implement physical activity recommendations in specific settings, as they have the necessary knowledge of contextual factors. However, dissemination and implementation of physical activity recommendations are often prevented by focusing exclusively on the health sector and by underestimating the individual perceptions and needs of change agents. Therefore, the purpose of this study was to address the problem of how physical activity recommendations can be translated into practice through comprehensive consideration of the situation and context of change agents from various sectors of society at different administrative levels. This allows for deriving recommendations for action on how a national dissemination strategy of physical activity recommendations should be designed.

**Methods:**

Qualitative expert interviews were conducted with change agents from different sectors of society and administrative levels in Germany (*N* = 21). Case selection took place via a sampling plan. The interviews were recorded, transcribed verbatim, and analysed by two trained researchers using qualitative content analysis.

**Results:**

The change agents’ perceived relevance of physical activity and physical activity promotion and their knowledge of physical activity recommendations varied across different sectors. Nine themes were identified covering the change agents’ needs for the implementation of physical activity recommendations: strengthening of political will and cooperation, availability of public space for physical activity, change in awareness and health education, professional qualification, financial incentives, development of physical activity-promoting programmes and structures, provision of resources, bridging the theory–practice gap, and knowledge of physical activity recommendations.

**Conclusions:**

This exploratory study contributes to the development of an evidence-based dissemination strategy of physical activity recommendations involving change agents from various sectors. Cross-sectoral needs and obstacles were identified indicating gaps that have to be addressed. Future research should choose practice-oriented approaches to develop dissemination strategies that are adapted to the needs of local contexts.

**Supplementary Information:**

The online version contains supplementary material available at 10.1186/s12961-021-00729-7.

## Background

Numerous studies have shown that regular physical activity (PA) and reduced sitting habits have a positive impact on physical and mental health for people of all ages [1]. However, PA recommendations are reached by only a small part of the population. In Germany, for example, only about a quarter of children and adolescents are sufficiently active [2]. The percentage is even lower in other parts of the world [3] and other age groups [4]. Lack of PA is not only responsible for 10% of all deaths [5] but is also associated with increased economic burden [6, 7].

Recently, the German Federal Ministry of Health published the German National Recommendations for Physical Activity and Physical Activity Promotion (NRPP) [8]. An update of the WHO guidelines on PA and sedentary behaviour was launched as well [9]. However, a common problem is that research findings on public health-related topics such as PA promotion are oftentimes not translated into practice, resulting in a research–practice gap [10–12]. Consequently, there is a strong need to develop strategies on how to bridge the gap and implement PA recommendations in specific settings [10, 12–14].

To change people’s PA behaviour and to implement PA recommendations, it is important to change the relevant environmental conditions. This is based on the socio-ecological paradigm which postulates that human behaviour takes place in interrelated and complex ecological systems [15–18]. The systems perspective acknowledges that people live, work, and learn in different multilayered environments (interpersonal, organizational, community, societal). These environmental conditions include social influences, such as social support or social norms, and structural influences, such as spatial conditions and available resources.

Environmental conditions are often under the control of change agents acting on different levels of the socio-ecological model: interpersonal (e.g., teachers), organizational (e.g., sports clubs administrators), community (e.g., urban planners), and societal (e.g., politicians at the national level). Based on theories of organizational change and development, different levels of change agents have to be considered [19]: Actors at a higher level can initiate other processes, such as exercising decision-making power and passing laws, than actors at a lower level, who usually have direct contact with target groups and use their professional skills in interacting with them.

Change agents may act as role models [13, 20], decision-makers [21, 22] and/or knowledge mediators [10]. According to the diffusion of innovations theory, the adoption and implementation of an innovation, such as PA recommendations, depends on the position of individual change agents in the innovation decision process, which is divided into five phases: knowledge of innovation, persuasion of innovation (positive or negative), decision for or against innovation, implementation of innovation, and confirmation of implementation decision [23].

Consequently, to implement PA recommendations by changing the environmental conditions, one must impact the change agents’ behaviour. The involvement of relevant stakeholders [12, 21, 24, 25], such as change agents [27, 28], is a common strategy to implement PA recommendations in specific settings, as they have the necessary knowledge of contextual factors. They can give valuable practical insights and draw attention to real-world challenges to develop measures that are adapted to local contexts [10, 12, 26, 29]. In addition to implementing concrete measures, change agents may also take on other roles in public policy processes [30, 31]. While actors with political decision-making power on a national level can influence agenda-setting, actors from the research community can participate in policy formulation [26, 29, 32].

However, dissemination and implementation of PA recommendation are often prevented by two failures: (1) Until now, they have neglected to involve all important sectors of society in PA promotion plans [33]. Although PA recommendations are often published by the health sector, their implementation is the responsibility of other sectors such as education, sport, and urban planning, which are generally rarely considered [29, 34, 35]. Additionally, socio-ecological models call for multilevel and multisectoral interventions to bring about a sustainable change in environments favourable to PA behaviour [16, 27]. However, until now, studies have often focused exclusively on change agents from the health sector [10, 26, 36] or only on individual settings such as schools [37] or the built environment [32]. (2) The individual perceptions, attitudes, and needs of change agents have not been sufficiently taken into account [33]. To enable change agents to promote PA and implement PA recommendations, health promoters and scientists should identify the factors that facilitate or hinder PA-promoting activities in change agents. This should include facilitators and barriers on the personal and environmental levels [27]. The survey of change agents’ needs allows for deriving recommendations for action with regard to which cross-population and setting-specific measures are necessary to implement PA recommendations. Needs cover demands of change agents that can be assigned to different levels of the socio-ecological model. In the following, a distinction is made between needs on the policy level and needs in certain behaviour settings. The former are defined as mainly indirect requirements aimed at changing policies concerning social and political issues. The latter refer to specific and personal demands in certain settings.

The purpose of this study is to address the problem of how national PA recommendations can be translated into practice through a comprehensive consideration of the situation and context of change agents promoting PA. There is a strong need to involve change agents in sectors of society other than the health sector and to focus on various settings when developing dissemination strategies for PA recommendations [36]. To identify the facilitators of and barriers to their behaviour, it is paramount to first survey the change agents’ perceived relevance of PA and PA promotion, their knowledge of PA promotion and PA recommendations, and their needs for implementing these recommendations.

Therefore, this study aimed to develop recommendations for action on how a national strategy for dissemination of PA recommendations including relevant change agents should be designed by (a) investigating the *change agents’ perceived relevance and knowledge* with regard to PA and PA promotion, and (b) analysing their *needs* with regard to the implementation of the NRPP in specific settings.

## Methods

### Study design

We chose an exploratory, qualitative approach, as little is known about this research area so far. The study took place in Germany, a country in central Europe with a population of about 83 million. Between October 2019 and April 2020, in-depth semi-structured expert interviews [38] were conducted to gain insight into the perceived relevance and knowledge as well as needs of change agents promoting PA. This approach is an adequate method to extensively record the participants' backgrounds, motivations, and explanations about a specific social phenomenon.

The interview guide included the following questions:

- What is the change agents’ perceived relevance of PA in society? How important is PA and PA promotion in the change agents’ organizations?
- What knowledge do change agents have regarding PA effects and PA promotion? Are they aware of the NRPP?
- Who are the change agents’ target groups?
- What are the needs for implementing the NRPP in the change agents’ specific settings?
- What are the problems and obstacles change agents encounter in implementing the NRPP?
- How do change agents assess their capabilities and potential for the implementation of the NRPP?

### Procedure and recruitment

The experts were selected based on existing and potential change agents of PA promotion in Germany identified in the SAMBA [Systematische Erfassung relevanter Akteure, Berufsgruppen sowie künftiger Multiplikatoren in der Bewegungsförderung zur Analyse und Entwicklung eines interdisziplinären Netzwerks zur nachhaltigen Bewegungsförderung] study [39]. In this study, different environmental conditions influencing individual PA behaviour were taken into account (interpersonal, organizational, community, societal), resulting in a compilation of change agents from a variety of sectors of society (politics and administration, health, sport/nonprofit, sport/for-profit, economy, media, education and research, social affairs) at different administrative levels (national level, state level, community level).

To ensure multisectoral representativity regarding PA promotion, we took the following sectors and change agents into consideration, which were derived from the SAMBA study [39]:

- Politics and administration: politicians, ministries, and departments at the national, state, and community levels; urban, transport, and landscape planning; health conferences at the state and community levels, etc.
- Sport/nonprofit: sports associations at the national and state levels, sports clubs, etc.
- Sport/for-profit: fitness industry, fitness centres, commercial sports providers, etc.
- Health: health insurance companies, occupational physicians, etc.
- Education and research: universities, colleges, schools, kindergartens, adult education centres, sport scientists, etc.
- Economy: sporting goods manufacturers, corporate health management providers, etc.
- Social affairs: churches, community welfare organizations, etc.
- Media: media, actors, etc.

The case selection took place via a sampling plan. For this purpose, the previous list was taken as a basis and expanded in certain areas. We deliberately aimed at covering different administrative levels (community, state, and national level) in all relevant sectors of society and thus also the different levels of the socio-ecological model [15, 16]. The participants were selected based on professional expertise. The expert status of the selected persons was discussed extensively within the project team prior to selection and contact to ensure that high-quality information could be generated. During the interviews, the experts repeatedly emphasized the importance of further actors, which were initially not considered in the sampling plan. Therefore, the plan was selectively expanded and additional experts were recruited. If an interview did not provide sufficient information on the specific sector of society, another expert from the relevant field was contacted.

The experts were recruited by email. In a cover letter, the participants received all relevant information regarding the background, content, and the planned procedure of the study. If participants did not answer, we followed up by telephone. Before the interview began, the experts were asked for their consent to the interview being recorded. They were also informed that their statements would be treated confidentially and made anonymous in the evaluation process. The interviews were conducted by two trained researchers.

### Study sample

A total of 21 expert interviews were conducted (19 by telephone and two face-to-face interviews). On average, an interview lasted approximately 42 minutes (range 19–73 minutes). In two cases, the interviews took place with two experts (e.g., two staff members of the same organization) at the request of the interview partners, resulting in a total of 23 experts. Seven of the respondents were female and 16 were male. Seventeen experts held management positions.

The composition of the experts with regard to the sectors of society and administrative levels was as follows (the respective *N* refers to the number of interviews, not to the number of experts):

- Politics and administration: national local-authority administration of cities, local department of urban planning (*N* = 2)
- Sport/nonprofit: national sports association, federal state sports association, local sports club (> 7000 members) (*N* = 3)
- Sport/for-profit: professional organization in the fitness industry, local fitness and health centre (*N* = 2)
- Health: health insurance company, occupational physician, primary care physician (*N* = 3)
- Education and research: national association of sport science, university (department of health science), school, kindergarten (*N* = 4)
- Economy: two sporting goods manufacturers, corporate health management provider (*N* = 3)
- Social affairs: church, city youth committee (*N* = 2)
- Media: national weekly news magazine, fitness and nutrition blogger (*N* = 2)

### Data analysis

The interviews were recorded, transcribed verbatim, and read several times. In the transcribed interviews, the interviewees were anonymized and were marked with the abbreviations “B1” to “B21”, and the interviewer with the abbreviation “I”. In the interviews with two interviewees at the same time, a and b were added as suffixes. For transcription, the f4transkript software package was used.

To evaluate the interviews, the MAXQDA software package was used. We conducted a computer-aided structured qualitative content analysis [40–42] with the aim of developing a category system to extract the relevant information to inform our study. While the main categories were deductively derived from the interview questions and therefore according to the research aims of this study, the subcategories were developed inductively drawing directly from the data material.

Since intercoder reliability is a critical component of qualitative content analysis, the transcripts were coded independently by two trained researchers in a circular process. Discrepancies were resolved by discussion. Based on intercoder agreement, 629 categories and subcategories were finally developed and interpreted. The definitions of the main categories were based on the following dimensions: perceived relevance of PA and PA promotion; knowledge of PA effects, PA promotion, and the NRPP**;** target groups of change agents; needs for the implementation of the NRPP**;** problems and obstacles to implementing the NRPP**;** and potential and capabilities as a change agent.

## Results

To answer the research questions, the six main categories developed were evaluated and summarized. Anonymized quotes from the interviewees are included as evidence. The information in brackets after each quote refers to the interview number, the respective paragraph of the interview, the sex of the interviewee, and the corresponding sector of society.

### Perceived relevance of PA and PA promotion

For the change agents interviewed, the topic of PA and PA promotion was of high to very high relevance. No one considered the topic to be unimportant. The significance of PA and PA promotion for physical and mental health was most frequently cited (*N* = 14), especially for the prevention of diseases and avoidance of medication:

*Physical activity and sports is actually the key to our health and is what everyone can do for themselves. On the one hand for physical health but also for mental health. […] [It] can also have the effect that each person can perhaps take less medication in his life.* (B6, 10, female, media)

Moreover, the effect of PA supporting social interaction and cohesion (*N* = 3)**,** the essential roles of PA in the motor development, socialization process, and holistic learning of children and adolescents (*N* = 4), and the importance of PA for the de-escalation of violence (*N* = 1) were emphasized. The economic relevance of PA promotion was mentioned by one change agent from the sport/for-profit sector.

More than half of the change agents surveyed rated the importance of PA and PA promotion in their occupation and organization as high (*N* = 11). Two change agents from the politics and administration sector and media sector assigned a medium value to the topic, as it was one of many issues their organization was dealing with. The change agents from the sporting goods industry and urban planning department indicated that PA promotion played no or only a subordinate role in their organization (*N* = 3). Although it had a high priority internally for one of the representatives of the sporting goods industry, as it was part of their employee health management, it was considered not relevant for their external strategy. The interviewee pointed out that they were primarily a company that produces clothing and not a nonprofit organization promoting PA. According to him, it was only conceivable to take up PA promotion within the context of a marketing campaign. The representative of the urban planning department also reported having little contact with the topic of PA promotion but rather saw the responsibility in other community departments:

*I actually have nothing to do with that, I mean the topic of physical activity promotion. Even regarding bicycle traffic, our focus is on overall urban mobility and the sustainability of traffic planning and not on health promotion.* (B20, 14, male, politics and administration)

The remaining five change agents could not be assigned to any of the three subcategories (high, medium, or low priority) as they presented a more differentiated view. While the primary care physician, for example, attached great importance to PA and PA promotion in her practice, she considered the importance in other medical practices as lower. The change agents from the school, the kindergarten, and the federal state sports association emphasized that the relevance of PA promotion was determined by the focus, orientation, and managerial staff of the individual organizations. According to the school representative, the importance of PA promotion depends mainly on how the school wants to advertise itself to the outside world to increase enrolment rates and is not intrinsically motivated.

### Knowledge of PA effects, PA promotion, and the NRPP

Thirteen of the change agents rated their knowledge of PA effects and PA promotion as very good, four as good, three as average, and one interviewee reported having no knowledge at all in this field. The ones who assessed their knowledge as very good had most commonly completed a university degree in PA and sport sciences. Further backgrounds of knowledge acquisition included practical and professional experience as well as further education and training. Eleven change agents were familiar with the NRPP, six of them were well acquainted with the content, one had basic knowledge, and four had only heard of the recommendations. The remaining 10 change agents (e.g., school, kindergarten, church, city youth committee, corporate health management provider) were unaware of the NRPP, but had already heard of the WHO PA recommendations (*N* = 5) or were unaware of any recommendations (*N* = 5).

### Target groups of change agents

Target groups of change agents include individual and collective actors. The most frequently named individual actors were cited with regard to different life phases (infants, children, adolescents, and seniors) or with regard to their roles (pupils, students, employees, and parents). As collective actors, the most frequently mentioned target groups were companies, federal state sports associations, and sports clubs. Change agents from the politics and administration, education and research, health, sport/nonprofit, and media sectors had a relatively broad target group, starting with children and adolescents, adults and employees, up to senior citizens. Target groups of change agents from other sectors were more specific. Change agents from the sport/for-profit sector most frequently named adults, employees, competitive athletes, and people with health problems, while change agents from the social affairs sector targeted primarily young and old people: children, adolescents, and senior citizens. Target groups of the economic sector included not only employees but also politicians, other companies, and sports clubs. The change agents were in contact with their target group partly directly (*N* = 10) and partly indirectly via mediating instances (*N* = 11). The latter were predominantly change agents who were located at higher administrative levels.

### Needs for the implementation of the NRPP

The needs of change agents regarding the dissemination and implementation of the NRPP were assigned to different environments (political, infrastructure, healthcare, workplace, sports and recreation, and information environment) and levels (policy level and behaviour setting level) of the socio-ecological model [16] (see Table 1). While policy-level needs are mainly indirect requirements aimed at social or political changes, needs of behaviour settings denote more specific and personal demands. In the following, the requirements are assigned to different themes that emerged in the course of the analysis.

**Table 1:** Needs of change agents regarding NRPP implementation structured according to environments and levels

Political environment	Infrastructure environment	Healthcare environment	Education environment	Workplace environment	Sports and recreation environment	Information environment
Policy level						
Political will and responsibility at national level / Agenda-setting PA promotion Establishment of a federal institution responsible for PA promotion Improvement of cooperation between ministries and political institutions Strengthening of political significance of PA and sport sciences	More public space for everyday-life PA and unorganized sport (ensure lighting and safety) Anchoring PA promotion in planning specifications of urban planners Political focus away from car traffic to more PA-friendly mobility concepts	Higher priority of PA promotion across spectrum of medical treatment Financial incentives for physicians and health insurance companies regarding preventive measures	Integrating PA promotion in vocational training of educational staff (schools, kindergartens, social institutions)	Increasing tax allowance for companies investing in employee PA promotion	Financial support for sports clubs engaging in PA promotion programmes Implementation of a quality seal for PA programmes	Change in society’s awareness of importance of PA and strengthening of society’s health literacy through nationwide media campaigns Publicizing NRPP to target groups, general population, and change agents Closer cooperation between science and practice
Behaviour setting level						
Empowerment of mayors and communities to implement the NRPP Improvement of cooperation between decision-makers at the state and community level	Change in awareness among urban planners regarding PA promotion	More attractive reward systems and financial resources for individuals engaging in PA Strengthening responsibility of physicians regarding PA promotion More personnel resources and time capacities	Change in awareness among educational staff regarding PA promotion High-quality and multifaceted physical education / Imparting of health competences Anchoring PA promotion in everyday school life Cooperation with parents regarding PA and active mobility More personnel and financial resources as well as time and spatial capacities Socialization of children towards PA	Change in organizational culture towards importance of PA (especially managers) Workplace PA programmes, PA-promoting infrastructure, flexible working hours More financial resources and time capacities	Establishment of new sports clubs focusing on PA promotion Offering PA programmes attractive to all age groups More public programmes within communities as well as digital PA programmes Health checkups to provide structured guidance	Practical information and working aids on how to implement the NRPP in specific settings More compact, up-to-date, and comprehensible (online) presentation of the NRPP

#### Strengthening of political will and cooperation

The majority of change agents emphasized the importance of increasing the political will and support towards disseminating the NRPP and the need for central coordination and responsibility for PA promotion on a national level (*N* = 14). The topic should take a higher priority on the political agenda, as one change agent framed it:

*One should really seriously put this issue of physical activity at the top of the agenda. If you look at scientific evidence, physical activity is so important that it is not high enough [on the political agenda]. This would be a first, very important step towards making it a top priority and ensuring that it is backed up by greater commitment and seriousness.* (B2b, 121, male, education and research)

According to two change agents (education and research, sport/nonprofit), the establishment of a national institution that is responsible for PA and PA promotion is necessary so that more personnel and financial resources are available at the political level. In addition, cooperation between existing federal ministries and political institutions as well as state- and community-level decision-makers involved in PA and PA promotion must be improved. One change agent, for example, considers it important to be better integrated into relevant networks:

*Well, we would have to cooperate in a different way. […] It’s a bit alarming that someone like me who is involved in this educational work didn’t even know about your publication. And I don’t know how the distribution works or how we could cooperate. It’s interesting that we are working on the same interfaces, on the same topics; therefore, we should form a better network, be in contact with each other.* (B5, 52, male, social affairs)

On the level of behaviour settings, the political responsibility for implementing the NRPP was seen to lie with the communities and mayors (*N* = 2; politics and administration, sport/nonprofit). They can directly influence the living environment, put both setting-specific and life stage-specific measures into practice, and should thus be empowered:

*After all, communities maintain a large number of institutions or even [...] community planning processes that are relevant in this area, whether it is community health planning, school development planning, and sports development planning, or urban development planning, where areas relevant for physical activity are also involved. […] This means that local authorities can transport this in their planning processes and through their institutions, they are responsible for kindergartens, they are responsible for schools, they are responsible for youth facilities, etc. […], and thus get relatively close to the people, to their neighbourhoods and where they live.* (B7, 58, male, politics and administration)

#### Availability of public space for PA

Concerning the infrastructure environment, the need for more public space for everyday-life PA and unorganized sport was emphasized (*N* = 5; diverse sectors). For one change agent, this is particularly important for young people:

*Adolescents are fighting for every single [sports opportunity] in their district. And I think that this should actually be more natural, that it should simply be available to young people. Also that you simply establish public spaces in the city centre that encourage physical activity.* (B4, 70, female, social affairs)

As a possible solution, a change in the planning specifications was mentioned, so that a certain percentage of the planned area may not be built on but must be available for public PA.

#### Change in awareness and health education

Nearly all change agents (*N* = 17) emphasized that a change in society’s awareness of the importance of PA and the provision of health education (*N* = 13) are required. For this purpose, the need for nationwide media campaigns promoting PA that involve a wide variety of media was stressed (*N* = 12): the use of social media and the promotion of PA via influencers was seen as particularly beneficial. Those campaigns should be centrally controlled by the federal government and involve different administrative levels:

*Such a pervasive movement from the government, from this ministry accordingly through the DOSB [German Olympic Sports Confederation] into the population. So, not only through the experts in the sports clubs but directly to the citizen without a club or anything else being interposed, that would of course be super desirable. TV spots, social media, radio spots, a really broad movement accompanied by motivational programmes.* (B18, 52, male, sport/nonprofit)

The need for a change in awareness to implement national PA recommendations was mentioned not only concerning the overall population but also at the level of specific behaviour settings. This referred to educational staff and the management responsible for schools, kindergartens or social institutions (*N* = 6; diverse sectors), employers and managerial staff within the workplace environment (*N* = 5; diverse sectors), and physicians and healthcare staff (*N* = 4; education and research, health, sport/for-profit). Even the change agent from the urban planning department stated that it is not enough to just change the planning specifications, but also the awareness among urban planners:

*But it is not yet in the minds of planners that the public space will be planned first and the parking lots and car traffic come second. In other words, the focus should be shifted away from the car and towards active mobility.* (B20, 42, male, politics and administration)

#### Professional qualification

Several change agents reported a need to better integrate PA and PA promotion into the vocational training of educational and healthcare staff, as these topics are currently underrepresented (*N* = 8; diverse sectors). Particularly with regard to schools, the need for sufficiently qualified teachers was emphasized, so that high-quality and multifaceted physical education including alternative ways of evaluation and lower levels of pressure to perform can support NRPP implementation in behaviour settings (*N* = 3; education and research, health). Moreover, all teachers should be capable of teaching health skills.

Some change agents argued that PA should become a larger part of the medicine course or further education of healthcare staff (*N* = 3; health, politics and administration). Physicians should become more aware of their function as role models and be able to make concrete recommendations to patients on how to increase PA behaviour (*N* = 4; education and research, health, sport/for-profit):

*I think that it would also be important that physicians are really qualified in the sector in a structured way and that they see the relevance for themselves and get involved. […] It is not enough just to say: “Do more sports”. It should be done with emphasis. […] And simply producing colourful brochures or placing a website is just not enough. It has to be a chain. That the person [physician] does not just say “Do more sports”, but also “Where exactly and how exactly and why does it work for you”. It would be desirable that there is more structure in the whole thing.* (B2b, 142, male, education and research)

#### Financial incentives

The requirement for financial incentives to persuade change agents to engage in NRPP implementation was mentioned several times. Some change agents stated that physicians and health insurance companies should receive more financial incentives for prevention and less for repair medicine (*N* = 4; diverse sectors). One change agent demanded that physicians be able to charge for prescribing PA:

*The problem which we get feedback on again and again is that it [prescribing PA] is still a voluntary service of physicians. Through the prevention law and through preventive medical checkups, it can be minimally charged for, but that is not what physicians want. So, it is an on-top service, and we find that in many federal states, sports physicians that are intrinsically motivated and have a sporting affinity use it very frequently, but with the average physician, there is still room for improvement. That is why we always demanded that it must be billable.* (B9, 104, male, sport/nonprofit)

Concerning the workplace environment, the need for increasing the tax allowance for companies investing in the promotion of employee health and PA was mentioned (*N* = 2; sport/nonprofit). Moreover, within the sports and recreation environment, sports clubs that explicitly engage in NRPP implementation should be financially supported according to one change agent (sport/nonprofit).

On the level of behaviour settings, the provision of more attractive reward systems and financial resources for individuals achieving the NRPP was emphasized, to persuade people to take more personal responsibility for their health (*N* = 6; diverse sectors). The use of sanctions was also discussed, so that behaviour harmful to health has a negative effect on, for example, the amount of health insurance contributions. However, for some interviewees, this is too great a violation of privacy.

#### Development of PA-promoting programmes and structures

Eleven change agents considered the development of specific PA programmes as useful for the implementation of the NRPP. While some stressed the importance of everyday exercise and fun-focused programmes, others demanded structured training programmes or called for new sports clubs that focus primarily on PA promotion. Even in existing sports clubs, the need for PA programmes for all age groups aimed at achieving the NRPP was emphasized (*N* = 4; education and research, sport/nonprofit):

*The problem I see in sports clubs is often that they do not have adequate and attractive exercise programmes for middle-aged people. […] And I think that there must be attractive offers for all target groups in organized sport on a really comprehensive basis aiming at the implementation of physical activity recommendations and not just sport-specific programmes. For children and adolescents, especially for children when they are small, there must be a wide range of programmes that promote all motor skills equally.* (B2a, 130, male, education and research)

To meet the trend towards increasing self-organized PA, some change agents emphasized the need for more digital programmes accessible to everyone (*N* = 5; diverse sectors) as well as public programmes within communities that are connected to the natural living environment (*N* = 4; education and research, health, politics and administration):

*There is already a trend towards physical activity; however, there is less willingness to commit oneself to any kind of sports club but more to the self-organized sport, which can be promoted by creating appropriate sports opportunities that have an inviting character.* (B7, 32, male, politics and administration)

One change agent (sport/for-profit) emphasized the policy-level need for developing a universally applicable quality seal so that high-quality PA programmes can be distinguished from lower-quality programmes to keep people from choosing PA programmes that are not suitable for them.

Apart from the development of PA programmes, the provision of PA-supporting structures in settings such as kindergartens, schools, and workplaces was seen as important. Seven change agents emphasized not only the availability of workplace PA programmes as a need, but also the provision of a PA-promoting infrastructure and flexible working hours. The same applies to schools, where PA programmes should be firmly anchored in every day’s schedule.

#### Provision of resources

To firmly anchor PA-promoting structures and programmes in diverse behaviour settings such as schools, kindergartens, workplaces, or doctors’ practices, the need for more resources was expressed. Several change agents from different sectors of society (especially from the education and research sector) called for more personnel and financial resources as well as time and spatial capacities to implement the NRPP in kindergartens and schools. The need for more personnel resources and time capacities was also mentioned by the primary care physician.

#### Bridging the theory–practice gap

Seven change agents (diverse sectors) pointed out a theory–practice gap and demanded a translation of the scientific findings of the NRPP into concrete dissemination strategies:

*We already have certain instruments and we have good structural conditions and these must now be used. And it is very important to make a transfer to practice and to really provide the practice with something useful and not just scientific findings that are reflected in some great publications with impact factor and so on. This is all-important for science but does little for practice and for the mission to get people moving.* (B9, 93, male, sport/nonprofit)

This would require closer cooperation between science and practice and higher participation of the target group when designing measures. One change agent from the economic sector expressed the need for practice-oriented scientific studies and communication of their results:

*Well, I would perhaps wish that scientific studies would look a little more into reality. To say, I’ll give you an example, […] if I do certain exercises for 5 minutes a day at my workplace, is that enough, or how often do I have to do it to compensate for something. […] Because if I know that something can be done in shorter units, then I could also better persuade employers to say, “Integrate it during the day”.* (B14, 58-62, male, economy)

Some change agents expressed the need for practical information such as methodological kits, working aids, practical instruments, and structures that support actors close to the target group, for example, in organizing daily routines of PA promotion in their setting but also in applying for PA projects or communicating PA recommendations adequately (*N* = 3; education and research, sport/nonprofit).

#### Knowledge of PA recommendations

The need for communicating the NRPP to the general population, specific target groups, and other change agents was repeatedly mentioned (*N* = 7; diverse sectors). It was pointed out that a more compact and comprehensible presentation of the most important points via a website would be necessary. The recommendations should also have an up-to-date appearance and be distributed via as many press mailing lists as possible, both referring to a new website and sending the recommendations directly:

*I believe that if you would present it a little differently, we would simply address it much more often. Our reporting would also refer to it much more often. To do so, you would have to pick out the key points, i.e., the message to the people out there, and put it together in a compact form. [...] So if you manage to distill the most important messages to the people out there as concretely but also as concisely as possible and also present them well on the Internet, then we can refer to them much better. We can link to it again and again, and yes, I think that would also make it much better known.* (B6, 48, female, media)

### Problems and obstacles to implementing the NRPP

Problems and obstacles to implementing the NRPP mentioned by the change agents concern the political, healthcare, education, and sports and recreation environment.

Within the political environment, federalism was seen as an obstacle, as it often prevents the implementation of centrally controlled measures and programmes (*N* = 1; sport/nonprofit). Five change agents (education and research, social affairs, sport/nonprofit) also saw the lengthy decision-making processes at various political levels as a hindrance when implementing measures to promote PA.

Concerning the healthcare environment, the situation of health insurance companies was seen as problematic, as they are in a competitive situation with each other. Thus, their focus would be on one-off marketing campaigns to recruit new members and not on sustainable implementation of the NRPP:

*It is simply also the false incentives for health insurance companies. On the one hand, they are in competition with each other. On the other hand, they are supposed to do preventive work, and you can see it in the workplace settings, […] that is where a health insurance company can also support people who were insured with a competing insurance company. And that of course [leads] to the fact that they […] do marketing measures there. In other words, they always focus on how to recruit new members. So they do measures that reach a lot of employees at once […]. That means that the whole thing goes in the wrong direction with such a false incentive. They should rather be given an incentive to implement these national recommendations.* (B14, 98, male, economy)

The change agent from the health insurance company took a different view and believed that they already integrate the NRPP sustainably in many areas. However, she also admitted that closer cooperation with the scientific community is necessary and that they need political backing and financial support from the taxpayer for the implementation of structural measures.

Obstacles identified in the medical field were linked to medical progress and the dominance of the pharmaceutical lobby and industry (*N* = 3; health, economy). Often, medication would be prescribed to alleviate symptoms rather than to prevent the cause of diseases by promoting PA, as framed by one change agent:

*One problem I see is that medical progress is so good, so if I can insert a stent after all, why should I do cardiovascular prevention? Well, repair medicine is great on the one hand, of course. I don't need to be physically active. It'll be repaired somehow. But humans are programmed to be active, and at some point in the evolutionary process something must have gone wrong*. (B16, 65, female, health)

Concerning the education environment, excessively rigid daily routines in kindergartens and the participatory approach to children’s daily planning were identified as problems (*N* = 2; education and research, sport/nonprofit):

*Another reason is that we have a participatory approach. So, the children can decide almost everything freely and individually. And there are always enough offers that have nothing to do with physical activity because there is simply a very broad spectrum. And that’s why you can say that the recommendation is perhaps fulfilled by children who are already very much interested in physical activity and always choose active programmes, but with the others… if they choose rather sedentary offers […] in the kindergarten, then most of the day is already over, and I don’t think they would somehow make up for the rest of the day.* (B8, 102, male, education and research)

In the school setting, the frequent cancellation of physical education classes and the fact that classes, especially in primary schools, would be taught by unqualified teachers were identified as obstacles to the implementation of the NRPP. The performance orientation in physical education would also leave little time for the explicit implementation of the NRPP (*N* = 4; diverse sectors). Overall, many expectations were placed on the education system and especially on the school environment and physical education classes. However, the interviewee from the school setting pointed out that physical education cannot meet all the demands that are not met by other subjects. Students’ social environment would often be neglected, and schools would have little influence on whether and how children are socialized to sport by their parents.

Regarding the sports and recreation environment, permanent pricing pressure was identified as a problem within the fitness industry, where PA offers have to be as affordable as possible. As a result, the qualification of the personnel would suffer, so that a sustainable orientation towards health and PA promotion is rarely given (*N* = 2; sport/for-profit, sport/nonprofit). Another obstacle emphasized by one change agent regarding organized sport was the focus of sports associations and clubs on competition and on talent identification and recruitment rather than on PA promotion:

*But I always emphasize that 90, 95, 98% of the people who volunteer [in sports clubs] do so basically because they love their sport: soccer, handball, whatever it is. And why should they suddenly want something that doesn't really benefit their sport at first sight*. (B21, 78, male, sport/nonprofit)

### Potential and capabilities as a change agent

The majority of the change agents assessed their potential and capabilities regarding the dissemination of the NRPP as positive. While some believed that they can contribute to raising awareness regarding the NRPP, providing knowledge about PA and PA promotion (*N* = 6; diverse sectors), and qualifying specialists in the field of PA promotion (*N* = 2; education and research), others make concrete suggestions regarding PA to the target group (*N* = 2; health, sport/for-profit), contribute to motivating them (*N* = 3; economy, education and research, media), act as role models (*N* = 2; health, media), or can influence the conditions for implementing the NRPP (*N* = 2; education and research, sport/nonprofit). As a prerequisite for this, however, some also emphasized that the needs mentioned must be met at various levels. Four change agents were sceptical about their capacity to disseminate the NRPP (economy, health, sport/for-profit, sport/nonprofit). Although the importance of physicians in promoting PA was frequently emphasized, the primary care physician interviewed assessed her potential for influencing the PA behaviour of her patients as limited. Three change agents (social affairs, health, sport/nonprofit) perceived their influence rather indirectly as a creeping process and as one of many factors that can influence the PA behaviour of their target group.

## Discussion

The purpose of this study was to address the problem of how national PA recommendations can be translated into practice involving change agents of PA promotion. Through a comprehensive consideration of their situation and context, we identified facilitators and obstacles that have to be considered in dissemination strategies. Recommendations for action are highlighted in italics. A summary of the recommended actions is provided in Additional file 1.

The first aim of this study was to investigate the change agents’ perceived relevance and knowledge concerning PA and PA promotion. Overall, PA and PA promotion were of high to very high perceived relevance to all change agents. Although scientific studies show that the economic relevance of PA is considerable [6, 7], only one change agent mentioned this as a motive for engaging in PA promotion. To strengthen the importance of PA promotion, especially at the political level, it seems necessary to focus more on the financial consequences of a lack of PA and to communicate the results of relevant studies to decision-makers.

In their occupation and organization, about half of the change agents assigned high priority to PA and PA promotion. The topic was of low relevance to the sporting goods manufacturers and the department of urban planning. Due to their high level of popularity and their marketing budget, sporting goods manufacturers, in particular, have great potential for the dissemination of PA recommendations [43]. Actors that are not directly involved in health and PA promotion often do not realize that they play a significant role in this context [29]. It is therefore important to *develop strategies on how to integrate such change agents from non-health sectors into networks of PA promotion*. Concerning sporting goods manufacturers, a decisive step would be to give prominence to the *economic advantages of an engagement in PA promotion*, for example, taking up the topic in a marketing campaign. To persuade stakeholders in urban and transport planning, it is important to *present the topic in a broader context*, such as emphasizing quality of life instead of PA promotion, and to focus on the *ecological relevance* of PA promotion, such as sustainable mobility and climate protection [33].

Some change agents delegated responsibility for disseminating PA recommendations to other authorities, emphasizing that the relevance of PA and PA promotion is not in their hands but depends heavily on political decisions and the focus and management of individual institutions. A lack of coordination, the absence of a strategic plan, and the failure to take responsibility are phenomena frequently observed in other countries as well, hampering the dissemination of national PA recommendations [44, 45]. At this point, it is important to *better involve change agents in dissemination strategies* and to appeal to their personal responsibility for implementing PA recommendations.

Although the majority of change agents rated their knowledge in the field of PA effects and PA promotion as good or very good, about half had not yet heard of the NRPP. Strikingly, this concerned some change agents from the educational, social, and workplace environment who have direct contact with target groups. It seems that there is an intuitive rather than a systematic approach to NRPP implementation in Germany. One of the next steps should be to *make the NRPP known across sectors*, with a special focus on change agents interacting directly with relevant target groups in order to bring more structure into the dissemination and implementation process.

The second aim of this study was to analyse the change agents’ needs with regard to the implementation of the NRPP in specific settings. To give NRPP dissemination a *higher priority on the political agenda*, the *establishment of a national authority responsible for PA promotion* is needed. Furthermore, there needs to be *closer cooperation and networking of relevant change agents at the national, state, and community levels* to tackle PA promotion collectively. This is in line with the demands of previous research to form intersectoral networks to solve complex health problems, such as physical inactivity, by combining core competencies and resources, creating synergies, and working more effectively on solutions involving different perspectives [39, 46–48]. Drawing on findings from research on network governance [49], a central institution for PA might act as an administrative unit that initiates and manages multisectoral networks of relevant actors at the national level. In this way, it could take the leadership role concerning the implementation and evaluation of PA promotion.

Within the infrastructure environment*, more public PA spaces are needed*. Here, urban and landscape planning play an important role. Not only should the *planning specifications be changed* regarding the design of PA-promoting environments, but there must also be a change in awareness so that urban planners become aware of their responsibility concerning the implementation of the NRPP.

PA and PA promotion should become a larger part of the *vocational training of educational and healthcare staff*, as these topics seem to be currently underrepresented. In particular, teaching staff need to be adequately qualified with regard to *high-quality and multifaceted physical education* in which health skills are taught.

Overall, *more financial incentives* should be provided for the dissemination of the NRPP with regard to different target groups. In the health sector, a *stronger focus on disease prevention* is required [50]. Physicians should be able to charge for the prescription of PA, and health insurance companies should be rewarded for realizing sustainable NRPP implementation. More attractive reward systems for individuals as well as financial incentives for sports clubs and companies to engage in PA promotion were also suggested. To ensure that more financial resources are available regarding PA promotion, again a national institute for PA that has the appropriate resources would be important.

To ensure that greater attention is paid to the topic of PA promotion, a *change in social norms and awareness as well as health education* are needed so that the importance of PA and sport is anchored in the consciousness throughout society but also in specific settings. This requires, among other things, a *clearly elaborated communication concept that is disseminated through media campaigns*, especially involving social media. However, it has to be taken into account that the effectiveness of stand-alone mass-media campaigns in PA promotion is still unclear. It seems more effective to embed such campaigns into broader multicomponent interventions [51]. Also, care should be taken to comprehensively cover all relevant content of PA recommendations and to develop different communication concepts for different target groups [52]. In addition, there should be a *comprehensible and compact online representation of national PA recommendations* which is easily accessible to everyone.

The findings show that there is disagreement as to whether structured training programmes or fun-focused PA in everyday life are more effective in implementing the NRPP. Among other things, this highlights the need for *concrete information, working aids, and methodological kits* that can support change agents in implementing suitable, scientifically based measures of PA promotion. It is essential for this purpose that change agents are addressed in a *transdisciplinary approach of science and practice* to translate the scientific findings of the NRPP into political implementation strategies, medical treatment strategies, and specific PA-promoting measures useful in practice tailored to the respective setting [53]. Such an approach could also lead to change agents taking more responsibility for the implementation of jointly developed programmes.

Besides the *provision of more attractive PA programmes for all age groups provided by sports clubs* and the *development of easily accessible public and digital PA programmes*, the implementation of *PA recommendations should be structurally anchored* in the settings where people live, learn, and work. This includes the *establishment of a PA-friendly organizational culture and flexible working hours* by employers as well as *PA breaks and programmes that are firmly anchored in the daily routine of educational institutions*, supported by the respective management.

Schools, kindergartens, and organized sport are considered central settings for PA promotion [8]. However, the respective change agents point out some problems that currently hamper the implementation of the NRPP, such as too rigid daily routines, a lack of staff qualifications, a lack of space (e.g., gymnasiums and swimming pools), the cancellation of physical education, the shortage of financial resources, and a lack of awareness on the part of kindergarten, school, and sports club administrators. In addition, performance orientation in physical education and sports clubs would leave very little room for more general approaches to PA promotion. These findings are also supported by existing literature [54, 55]. In this context, it is even more important to *appeal to the personal responsibility of sports clubs and educational institutions* to critically question their performance orientation and to better fulfil their educational and social mission [54, 56–58].

To solve obstacles to the dissemination and implementation of PA recommendations in settings such as educational institutions, the workplace, or the healthcare setting, *appropriate resources have to be provided*. These can ensure that the personnel, time, and spatial capacities needed to carry out adequate PA promotion are covered.

**Fig. 1:**
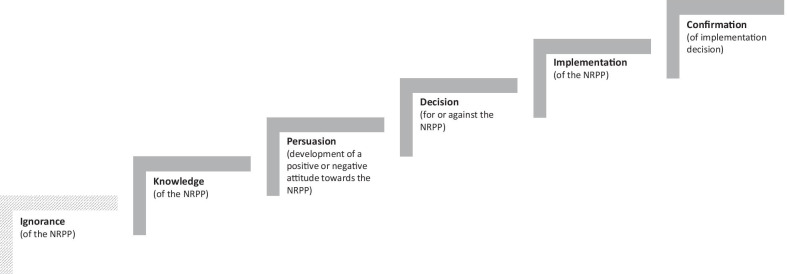
Innovation decision process towards NRPP implementation (modified according to Rogers [23])

Referring back to diffusion of innovations theory [23], one could assign the change agents and their needs to the different phases of the innovation decision process (Fig. 1). Change agents have different needs depending on the stage they are in, and therefore they have to be addressed accordingly to successfully involve them in the dissemination and implementation of PA recommendations. Since some change agents had not yet heard of the NRPP or even had no connection to the topic of PA promotion, such as the representative of the urban planning department, we added a further phase to the model: the ignorance phase. Change agents in this phase need to be informed about PA recommendations and their applicability in the first place. In addition, they should become aware that PA promotion is a relevant topic to them so that they move on to the knowledge and persuasion phase. One of the sporting goods manufacturers was convinced that the NRPP and PA promotion are something worth striving for but had not yet decided to engage in NRPP dissemination, which is why he could be assigned to the persuasion phase. To move on to the decision phase, change agents must be made aware of the advantages of an engagement in the dissemination of PA recommendations, for example, by *focusing on the economic, societal, or ecological benefits*. Change agents in the decision phase, such as the primary care physician, were willing to implement the NRPP but had not yet proceeded systematically. At this stage, appropriate financial resources, working aids, and practicable information that support change agents in implementing PA recommendations are needed. Some change agents (e.g., from the fitness and health centre or the health insurance company) could be assigned to the implementation phase, since they use the NRPP in a structured way in their daily work. These change agents need to be supported through appropriate resources and political backing to keep their implementation decision valid.

The study findings show that on the one hand, change agents need to take on more responsibility for implementing PA recommendations. On the other hand, for change agents to become active at all, certain requirements, such as agenda-setting or provision of resources, must be met at a higher level so that environments and conditions are favourable to this dynamic. Therefore, both the involvement of change agents and the whole organization of the system are determinants for the dissemination of PA recommendations.

The major strength of this study is the consideration of needs on the policy and behaviour setting level going beyond individual sectors of society: The present study is one of the first to involve stakeholders from various sectors of society and administrative levels in the development of a national dissemination strategy of PA recommendations. Furthermore, extensive data material was collected and analysed, ensuring high credibility of the study findings. Transferability was guaranteed through a thick description of contextual conditions and participants surveyed. Finally, intercoder reliability and self-reflection of the researchers during the research process ensured dependability and confirmability of the study findings. However, some limitations have to be mentioned: The study findings cannot be readily generalized. The change agents’ statements are based on subjective opinions, depending on their individual situation and context. Thus, some of the needs cited might be linked to predominant conditions (organizational structure, relevance of PA promotion, financial, spatial, and personnel capacities) of specific settings. Moreover, the majority of respondents were male. A greater diversity of interviewees and a more balanced ratio of women and men may have led to additional or different needs related to NRPP dissemination. To verify whether these findings are representative and applicable to other organizations of the same type, in-depth needs analysis of individual settings are necessary. Finally, the aspect of social desirability based on the interview situation must be taken into account. When asking the respondents about their motives for an engagement in PA promotion, altruistic motives such as health promotion may have been given preference over, for example, economic motives.

## Conclusions

This study enables a differentiated consideration of the perceived relevance, knowledge, and needs of change agents concerning the dissemination and implementation of the NRPP in Germany. Change agents, acting as decision-makers, knowledge mediators, and role models, from diverse sectors of society were taken into consideration. To our knowledge, this is the first study which covers different administrative levels and all sectors of society relevant to PA promotion. This exploratory study is an important step towards developing an evidence-based strategy for dissemination of PA recommendations involving various change agents of PA promotion. Even though each of the sectors under consideration has different goals and a more or less close connection to the topic of PA promotion, many cross-sectoral needs and obstacles were identified. They indicate gaps that need to be addressed and closed in dissemination strategies for PA recommendations. Taking into account their attitudes toward PA promotion and their needs regarding the implementation of PA recommendations, change agents and their respective environments should be addressed accordingly to engage them most effectively. Future research should identify the needs of specific settings in a representative way and develop concrete measures on how to involve change agents in the dissemination and implementation of PA recommendations. It is particularly important to choose transdisciplinary approaches of research and practice so that measures are adapted to the needs of local contexts. As PA promotion is a challenge that needs to be tackled by multisectoral cooperation, the study of the interactions of change agents on different levels might produce valuable insights regarding collaborative policy formulation and implementation. In addition, an evaluation of the success of policy development and dissemination strategies involving change agents in Germany and other countries is needed.

## Supplementary Information

**Additional file 1.** Recommendations for action.

## Data Availability

The datasets used and analysed during the current study are not publicly available for privacy reasons but are available from the corresponding author on reasonable request.

## Abbreviations

PA: Physical activity
NRPP: National Recommendations for Physical Activity and Physical Activity Promotion

## References

[1] Reiner M, Niermann C, Jekauc D, Woll A (2013). Long-term health benefits of physical activity: a systematic review of longitudinal studies. BMC Public Health.

[2] Schmidt SCE, Anedda B, Burchartz A, Oriwol D, Kolb S, Wäsche H (2020). The physical activity of children and adolescents in Germany 2003–2017: the MoMo-study. PLoS ONE.

[3] Aubert S, Barnes JD, Abdeta C, Nader PA, Adeniyi AF, Aguilar-Farias N (2018). Global Matrix 3.0 physical activity report card grades for children and youth: results and analysis from 49 countries. J Phys Act Health..

[4] Krug S, Jordan S, Mensink GBM, Müters S, Finger J, Lampert T (2013). Körperliche Aktivität: ergebnisse der studie zur gesundheit erwachsener in Deutschland (DEGS1). Bundesgesundheitsblatt Gesundheitsforschung Gesundheitsschutz.

[5] Hamer M, O'Donovan G, Murphy M (2017). Physical inactivity and the economic and health burdens due to cardiovascular disease: exercise as medicine. Adv Exp Med Biol.

[6] Centre for Economics and Business Research, International Sport and Culture Association. The economic cost of physical inactivity in Europe: an ISCA/Cebr report. 2015. https://inactivity-time-bomb.nowwemove.com/report/. Accessed 18 Apr 2020.

[7] Ding D, Lawson KD, Kolbe-Alexander TL, Finkelstein EA, Katzmarzyk PT, van Mechelen W (2016). The economic burden of physical inactivity: a global analysis of major non-communicable diseases. The Lancet.

[8] Rütten A, Pfeifer K (Hg). Nationale Empfehlungen für Bewegung und Bewegungs-förderung. Erlangen: FAU Erlangen-Nürnberg; 2016.

[9] World Health Organization. Who guidelines on physical activity and sedentary behaviour. 2020. https://www.who.int/teams/health-promotion/physical-activity/developing-guidelines-on-physical-activity-and-sedentary-behaviour. Accessed 27th November 2020. 33369898

[10] Ballew P, Brownson RC, Haire-Joshu D, Heath GW, Kreuter MW (2010). Dissemination of effective physical activity interventions: are we applying the evidence?. Health Educ Res.

[11] Brownson RC, Ballew P, Dieffenderfer B, Haire-Joshu D, Heath GW, Kreuter MW (2007). Evidence-based interventions to promote physical activity: what contributes to dissemination by state health departments. Am J Prev Med.

[12] Davis SM, Cruz TH, Kozoll RL (2017). Research to practice: implementing physical activity recommendations. Am J Prev Med.

[13] Bauman AE, Nelson DE, Pratt M, Matsudo V, Schoeppe S (2006). Dissemination of physical activity evidence, programs, policies, and surveillance in the international public health arena. Am J Prev Med.

[14] Pollack KM, Schmid TL, Wilson AL, Schulman E (2016). Advancing translation and dissemination research and practice through the physical activity policy research network plus. Environ Behav.

[15] Kok G, Gottlieb NH, Commers M, Smerecnik C (2008). The ecological approach in health promotion programs: a decade later. Am J Health Promot.

[16] Sallis JF, Cervero RB, Ascher W, Henderson KA, Kraft MK, Kerr J (2006). An ecological approach to creating active living communities. Annu Rev Public Health.

[17] McLeroy K (2006). Thinking of systems. Am J Public Health.

[18] Trochim WM, Cabrera DA, Milstein B, Gallagher RS, Leischow SJ (2006). Practical challenges of systems thinking and modeling in public health. Am J Public Health.

[19] Butterfoss FD, Kegler MC, Francisco VT, Glanz K, Rimer BK, Viswanath K (2008). Mobilizing organizations for health promotion: theories of organizational change. Health behavior and health education: theory, research, and practice.

[20] Babey SH, Wolstein J, Diamant AL (2016). Adolescent physical activity: role of school support, role models, and social participation in racial and income disparities. Environ Behav.

[21] Rütten A, Abu-Omar K, Messing S, Weege M, Pfeifer K, Geidl W (2018). How can the impact of national recommendations for physical activity be increased? Experiences from Germany. Health Res Policy Sys.

[22] Dobbins M, Robeson P, Ciliska D, Hanna S, Cameron R, O'Mara L (2009). A description of a knowledge broker role implemented as part of a randomized controlled trial evaluating three knowledge translation strategies. Implement Sci.

[23] Rogers EM (2003). Diffusion of innovations.

[24] Latimer-Cheung AE, Rhodes RE, Kho ME, Tomasone JR, Gainforth HL, Kowalski K (2013). Evidence-informed recommendations for constructing and disseminating messages supplementing the new Canadian Physical Activity Guidelines. BMC Public Health.

[25] Muellmann S, Steenbock B, de Cocker K, de Craemer M, Hayes C, O'Shea MP (2017). Views of policy makers and health promotion professionals on factors facilitating implementation and maintenance of interventions and policies promoting physical activity and healthy eating: results of the DEDIPAC project. BMC Public Health.

[26] Piercy KL, Dorn JM, Fulton JE, Janz KF, Lee SM, McKinnon RA (2015). Opportunities for public health to increase physical activity among youths. Am J Public Health.

[27] Bartholomew Eldredge LK, Markham CM, Ruiter RAC, Fernández ME, Kok G, Parcel GS (2016). Planning health promotion programs: an intervention mapping approach.

[28] Simons-Morton DG, Simons-Morton BG, Parcel GS, Bunker JF (1988). Influencing personal and environmental conditions for community health: a multilevel intervention model. Fam Community Health.

[29] van Rinsum CE, Gerards SMPL, Rutten GM, van de Goor IAM, Kremers SPJ (2017). Health brokers: how can they help deal with the wickedness of public health problems?. Biomed Res Int.

[30] Brewer GD, DeLeon P (1983). The foundations of policy analysis.

[31] Lasswell HD (1956). The decision process: seven categories of functional analysis.

[32] Giles-Corti B, Sallis JF, Sugiyama T, Frank LD, Lowe M, Owen N (2015). Translating active living research into policy and practice: one important pathway to chronic disease prevention. J Public Health Policy.

[33] Leone L, Pesce C (2017). From delivery to adoption of physical activity guidelines: realist synthesis. Int J Environ Res Public Health.

[34] Haggis C, Sims-Gould J, Winters M, Gutteridge K, McKay HA (2013). Sustained impact of community-based physical activity interventions: key elements for success. BMC Public Health.

[35] Woolf SH, Purnell JQ, Simon SM, Zimmerman EB, Camberos GJ, Haley A (2015). Translating evidence into population health improvement: strategies and barriers. Annu Rev Public Health.

[36] Brownson RC, Ballew P, Brown KL, Elliott MB, Haire-Joshu D, Heath GW (2007). The effect of disseminating evidence-based interventions that promote physical activity to health departments. Am J Public Health.

[37] Cooper KH, Greenberg JD, Castelli DM, Barton M, Martin SB, Morrow JR (2016). Implementing policies to enhance physical education and physical activity in schools. Res Q Exerc Sport.

[38] Gläser J, Laudel G (2004). Experteninterviews und qualitative Inhaltsanalyse.

[39] Wäsche H, Peters S, Appelles L, Woll A (2018). Bewegungsförderung in Deutschland: akteure, strukturen und netzwerkentwicklung. B & G.

[40] Elo S, Kyngäs H (2008). The qualitative content analysis process. J Adv Nurs.

[41] Mayring P. Qualitative content analysis. Forum: qualitative social research. 2000;1(2):20. https://www.qualitative-research.net/index.php/fqs/article/view/1089/2386 [Accessed 9th March 2021].

[42] Williamson K, Given LM, Scifleet P, Williamson K, Johanson G (2018). Qualitative data analysis. Research methods: information, systems, and contexts.

[43] World Health Organization (2004). Global strategy on diet, physical activity, and health.

[44] Bornstein DB, Pate RR, Pratt M (2009). A review of the national physical activity plans of six countries. J Phys Act Health.

[45] Spence JC, Faulkner G, Bradstreet CC, Duggan M, Tremblay MS (2015). Active Canada 20/20: a physical activity plan for Canada. Can J Public Health/Revue Canadienne Santé Publique.

[46] World Health Organization (2018). More active people for a healthier world: global action plan on physical activity 2018–2030.

[47] Bevc CA, Retrum JH, Varda DM (2015). Patterns in PARTNERing across public health collaboratives. Int J Environ Res Public Health.

[48] Provan KG, Milward HB (2001). Do networks really work? A framework for evaluating public-sector organizational networks. Public Adm Rev.

[49] Provan KG, Kenis P (2007). Modes of network governance: structure, management, and effectiveness. J Public Adm Res Theory.

[50] Vuori IM, Lavie CJ, Blair SN (2013). Physical activity promotion in the health care system. Mayo Clin Proc.

[51] Brown DR, Soares J, Epping JM, Lankford TJ, Wallace JS, Hopkins D (2012). Stand-alone mass media campaigns to increase physical activity: a Community Guide updated review. Am J Prev Med.

[52] Maddock JE, Kellstedt D (2020). Initial mass media coverage of the 2nd edition of the physical activity guidelines for Americans. Prev Med Rep..

[53] Glasgow RE, Emmons KM (2007). How can we increase translation of research into practice? Types of evidence needed. Annu Rev Public Health.

[54] Hills AP, Dengel DR, Lubans DR (2015). Supporting public health priorities: recommendations for physical education and physical activity promotion in schools. Prog Cardiovasc Dis.

[55] Skille EÅ (2010). Competitiveness and health: the work of sport clubs as seen by sport clubs representatives—a Norwegian case study. Int Rev Sociol Sport.

[56] Geidne S, Kokko S, Lane A, Ooms L, Vuillemin A, Seghers J (2019). Health promotion interventions in sports clubs: can we talk about a setting-based approach? A systematic mapping review. Health Educ Behav.

[57] Kokko S (2014). Sports clubs as settings for health promotion: fundamentals and an overview to research. Scand J Public Health.

[58] Lozano-Sufrategui L, Pringle A, Zwolinsky S, Drew KJ (2020). Professional football clubs’ involvement in health promotion in Spain: an audit of current practices. Health Promot Int.

