# Supplementary material for: How to disseminate national recommendations for physical activity: a qualitative analysis of critical change agents in Germany
Source: Health Res Policy Syst. 2021 May 6;19:78. doi: 10.1186/s12961-021-00729-7 (PMC8101134; doi:10.1186/s12961-021-00729-7)
Supplement: Supplementary file 1 — Additional file 1. Recommendations for action. [file 12961_2021_729_MOESM1_ESM.docx]

**Recommendations for action**

The aim of this study was to develop recommendations for action on how a national dissemination strategy of physical activity (PA) recommendations including relevant change agents should be designed by considering the *change agents’ perceived relevance and knowledge* concerning PA and PA promotion and their *needs* with regard to the implementation of the German National Recommendations for Physical Activity and Physical Activity Promotion (NRPP) in specific settings.

Based on the study findings, the following recommendations can be made:

- Strengthening the political significance of PA promotion by establishing a national institute for PA that centrally coordinates and is responsible for disseminating the NRPP.
- Changing awareness regarding the importance of PA in the population as a whole and in individual areas of society (politics, education, health care, workplace) through a clearly developed communication concept including media campaigns and health education.
- Improving the cooperation of relevant change agents at the national, state, and community level through the nomination of central coordinating administrative units (network governance).
- Appealing to the personal responsibility of change agents to engage in NRPP implementation in their respective setting and organization.
- Focusing on the economic, social, and ecological relevance of PA promotion to create incentives for change agents not involved in health (e.g., urban planning departments, sporting goods manufacturers) to implement the NRPP.
- Improving the cooperation between science and practice through transdisciplinary approaches to translate the scientific findings of the NRPP into political implementation strategies, medical treatment strategies, and specific PA promoting measures useful in practice.
- Communication of the NRPP to relevant change agents to enable a systematic approach to PA promotion. Compact online availability of the NRPP.
- Development and communication of methodological kits, working aids, and practical instruments that support change agents in a scientifically based and efficient implementation of the NRPP.
- More financial incentives for individuals, physicians, health insurance companies, employers, and sports clubs to become involved in PA promotion.
- Integration of PA promotion in the vocational training of teachers, kindergarten teachers, and social education workers. In particular, adequate qualification of teaching staff concerning high-quality and multi-faceted physical education, in which health skills are taught.
- Structural anchoring of PA promotion in educational institutions through PA offers and PA breaks.
- Establishment of a PA-friendly organizational culture, PA-promoting structures, and more flexible working hours by employers.
- Increase of financial, time, personnel, and spatial resources in educational institutions, workplace settings, and within the health sector.
- Availability of more public space for leisure sports and everyday PA through a change in planning specifications of urban planning departments.
- More attractive PA programs for all age groups provided by sports clubs and development of a broader range of easily accessible public and digital PA programs.
